# Sheltering difference: (un)doing the migrant/volunteer divide through sheltering practices in Mexico and the Netherlands

**DOI:** 10.3389/fsoc.2023.1084429

**Published:** 2023-05-18

**Authors:** Cesar E. Merlín-Escorza, Joris Schapendonk, Tine Davids

**Affiliations:** ^1^Department of Geography, Planning and Environment, Nijmegen School of Management, Radboud University, Nijmegen, Netherlands; ^2^Department of Cultural Anthropology and Development Studies, Social Sciences Faculty, Radboud University, Nijmegen, Netherlands

**Keywords:** sheltering practices, difference, interconnectedness, de-migranticization, conviviality, categorization

## Abstract

While acknowledging the important role of shelter organizations in protecting migrant rights, recent debates point to the thin line between care and control practices within shelters. This study seeks to deepen this observation by approaching shelters as spaces defined by a constant inward/outward mobility of people. From this starting point, we use the de-migranticization framework to understand and question the normalization of difference that divides migrant people (being reproduced as the typical guest) from international volunteers (being reproduced as the typical host) through sheltering practices in two rather different geopolitical contexts (Mexico and the Netherlands). We use our ethnographic insights to not only illustrate how difference is reproduced but also to analyze the practices that seek to transgress and undo these divides. We argue that highlighting the conviviality and interconnectedness between these differentiated actors in the broader context of cross-border mobility is of vital importance to question and overcome the coloniality of contemporary border regimes. However, we do not imply that these aspects have the potential to completely undo difference, as they are a constant struggle embedded in the relational practices of the people composing such a divide.

## 1. Introduction

Migrant shelters play a crucial role in people's erratic migratory processes across violent borders (Olayo-Méndez et al., 2014; Jones, 2017). They form important humanitarian infrastructures of protection for people who often find themselves outside the protection of the state. Migrant shelters are also important points for re-energizing people's journey, social networking, and emotional support. In this sense, migrant shelters in different places in the world function as important counter-hegemonic infrastructures in relation to strict border regimes. At the same time, and without disregarding their crucial role for people on the move, shelters are often discussed in academia in relation to the notion of humanitarianism (e.g., Sandri, 2018; Gomez et al., 2020; see also: Cuttitta, 2018). Several studies indicate that the practices of care by shelters do merge with the practice of control and discipline (e.g., Ticktin, 2011, 2016). In that sense, shelters indeed house their bordering dynamics (Angulo-Pasel, 2022). As a consequence of this care/control function, a particular imaginary of the prototypical migrant—as someone always in need, as someone vulnerable and agency-less, and as someone inherently different—tends to be reproduced over time. This study aims to delve further into the issue of how these social boundaries are made and unmade through practices of sheltering. This is particularly important since we have noticed that sheltering practices may in fact perpetuate migration management logics (Merlín-Escorza et al., 2021). This study builds on discussions around the humanitarian dimension of shelters and positions the question of sheltering within the so-called reflexive turn in migration studies.

The reflexive turn in migration studies centers around the question of how migration-related differences are produced and maintained in societal and academic knowledge production. One of the leading questions is how some cross-border mobilities are *turned into* particular forms of migration (Schapendonk, 2020; Amelina, 2021), while other similar mobilities are not labeled as such. Hence, this study addresses the problem of how migration-related differences are produced, particularly in the case of the fixed categorization of “the migrant,” placed in relation to the person meant to assist him/her/them referred as “the volunteer.” To analyze and contest such production of difference, we first attempt to de-migranticize (Dahinden, 2016) our analysis of the shelter. This implies that we seek to contest what seems to “divide” the people embodying such categories. This analytical step is crucial to *re*-politicize and fundamentally question certain taken-for-granted markers of difference that might be rather in line with the agendas of border regimes instead of being a true contestation of the same. From there, we analyze moments, situations, and practices where sheltering articulates migration-related differences as well as moments, situations, and practices that naturally overcome the same. Our analysis may not only fuel discussions on reflexivity in migration studies, but it ideally also informs the sheltering practices themselves, examining the extent to which these are part of the relentless fixation of bodies situated at border regions, in categories such as “the migrant,” “the asylum seeker,” “the undocumented,” and “the refugee.” Out-of-norm bodies, contained and objectified by state and academic migration apparatuses via the migranticization of their lives and mobilities (Dahinden, 2016), are differentiated according to the same logic that has historically legitimized the conception of borders and the state's sovereign power to exclude racialized non-national subjects, based on their (de)humanization (Mbembe, 2003; Lugones, 2010; Walia, 2013; Achiume, 2019). With this critical standpoint, we aim to contribute to this Special Issue (Ryan et al., this issue) by not only advancing knowledge on sheltering practices at the border and the relational politics involved but also by reflecting on the empirical and ethical challenges related to this research. In this process, we rely on the so-called methodological backstage approach (Aparna et al., 2020), which articulates the importance of scholarly reflexivity in questions of borders, emotional labor, and power asymmetries in fieldwork practices.

This study is based on long-standing ethnographic engagements with two shelter organizations being embedded in very different geo-political settings: southern Mexico and the Netherlands. The shelter organization in Mexico called *Casa para Todes* (House for Everyone)^1^, is embedded in a violent landscape of undocumented migration (Vogt, 2013; Estévez, 2014) historically shaped by the United States migration policy. In general, shelters in Mexico work as important stepping stones for people's trajectories, and they form a networked infrastructure for people on the move (e.g., Marchand, 2020; Wurtz, 2020). The shelter organization in the Netherlands called *Iedereen Welkom* (Everyone Welcome) is embedded in a typically Western European welfare-state model. However, especially in the last decade, the socio-political climate of shelter organizations is characterized by austerity politics. In contrast with the Mexican case, it is often considered that shelters in the Netherlands work with people at the end of a migratory trajectory. Such a narrative is instrumentalized by the state by integrating these kinds of non-governmental organizations into a deportation continuum (Kalir and Wissink, 2016), seeking to reinforce the already restrictive access to asylum in Europe. However, as we will see, the sheltering dynamics in place are often more complex.

The following sections are written mostly in first person from the first author's voice, as most of the ethnographic material comes from his experience. However, the second and third authors are within the “we,” constantly referred to throughout the text. Our argument does not place “the migrant” or “the volunteer” voices at the center, it rather highlights the relational processes and performativities in which these actors are embedded, instead of their personal views and narratives as isolated subjects. We start by discussing our analytical approach to sheltering practices, categorization as processes for differentiation, and the de-migranticization of the migrant/volunteer divide. Then, the methodological choices and ethnographic approach are explained. Subsequently, we use ethnographic material to depict the shelters' daily practices in three empirical sections. First, we start with our attempt to de-migranticize the narrative by looking at people's motives, interests, and geopolitical privilege defining their trajectories. This leads to a lexicon of *people looking for shelter* and *people looking to shelter*. This is not trivial as the usual divide of “migrants” vs. “volunteers” hides several particularities involved, such as the fact that volunteers do often have a history of migration themselves. Second, we look at the practices that articulate differences. Here, we discuss the shelter's intake process and the effects of linguistic plasticity in the use of labels. Finally, we show how the migrant/volunteer divide is transgressed and momentarily undone. Our conclusions draw on the reflections regarding our main findings, opening the question of how we—as academics—can learn from the sheltering practices under study?

## 2. Sheltering as doing migration: an analytical lens

Shelters have been understood as places where housing and humanitarian response are provided, especially in emergency contexts of displacement (Colosio, 2020), but also as processes rather than objects (e.g., Davis, 1978). Recent efforts to nuance the definition of shelter, propose “sheltering” as “an enabled process to facilitate a living environment with crisis-affected communities and individuals, to meet their current and future needs” (George et al., 2022, p. 12). Thus, understanding sheltering as a process, shelters as living environments where “guests” and “hosts” intermingle, and the goal of shelters to satisfy both actors' current and future needs are prominent elements in our analysis.

A fundamental aspect of sheltering processes at the organizations presented in this study is the differentiation made between people addressed as “migrants” and “volunteers,” and we refer to this as the migrant/volunteer divide. We consider such a divide to be related to modernity's categorization practices that are so dominantly present in questions of migration (Crawley and Skleparis, 2018). With different articulations—from border imperialism (Walia, 2013) to departheid (Kalir, 2019)—different authors stress the deep coloniality involved in categorizations by migration regimes (see also Lugones, 2010; Amelina, 2022; Wemyss, 2023; in this special issue). As argued elsewhere (Merlín-Escorza et al., 2021), we position the sheltering practices “on the ground” as an inherent aspect of the global migration governance architecture (van Riemsdijk et al., 2021), instead of practice outside that domain. Shelters, in other words, are not underground initiatives that destabilize the logics of borders and migration apparatuses. Instead of “un-making the border” (e.g., Peterson, 2020; Sandberg and Andersen, 2020), these shelters exist because of the border logic. However, in our view, that does not mean that shelter organizations resemble Goffman's (1961) notion of total institution. Although we do recognize some elements of discipline in our study, the sheltering practices discussed below are less absolute and less stable than Goffman's concept.

We aim to highlight the “politicizing function of the ‘naming' and ‘renaming' of categories of migrant mobility and experience” (Robertson, 2019, p. 229). In doing so, we depart from Amelina's (2021, 2022) notion of “doing migration,” which “refers to all social practices that, being linked to specific categorizations and narratives of belonging, membership and deservingness (i.e., discursive knowledge), turn mobile (and often also immobile) individuals into ‘migrants”' (Amelina, 2021, p. 2). Amelina, thus, proposes to analyze the social practices distinguishing “migrants” from “non-migrants” at institutional, organizational, and interactional levels. This approach helps to understand the behaviors associated with each category, in relation to classifications based on gender, ethnicity, race^2^, class, space, and other “categories of inequality” (Amelina, 2021, p. 3). At the interactional level of shelter organizations, multiple narratives making distinctions between us and them are performed on daily face-to-face routines. Although such narratives indeed subject people to “migrant” or “volunteer” positions, the previous analysis showed that people also perform multiple narratives using “floating signifiers” to negotiate their positionality and to better navigate the shelter's power dynamics (Laclau and Mouffe, 1985; Merlín-Escorza et al., 2021). Our interest is to focus on the mechanisms that facilitate these transitions or *passings* between narratives and performativities of “migrant” and “non-migrant” at the shelters' interactive level.

For this purpose, we follow Janine Dahinden's suggestion to “de-migranticize” migration studies (Dahinden, 2016). Similarly to Amelina, Dahinden points out the discursive normalization of categories such as “migrant” and “refugee” by the migration apparatus of nation-states and academia, specifically referring to migration and integration scholars. For her, the problem lies in the way in which migration research is marked by an epistemology that normalizes “migration- and ethnicity-related difference,” being the “national-container” logic of inclusion and exclusion, “the most important reference system for empirical research and theories” (Dahinden, 2016, p. 2,209). To move beyond such normalization, Dahinden proposes to re-orient the focus of investigation away from “migrant populations” toward overall populations and distinguish common-sense categories from analytical ones (Dahinden, 2016).

We combine Amelina and Dahinden's proposals to analyze different sheltering practices. We focus on the im/mobility of “entire populations” related to the shelter, instead of focusing on the exceptionalized movements of the “migrant other” only, doing so by juxtaposing the motives, interests, and needs of differentiated populations in sheltering practices. Here, we are inspired by Malkki's (2015) work on Finnish Red Cross voluntary aid workers, where she questions the “basic assumptions about who *the needy* are in the humanitarian encounter,” and how subjectivities producing the humanitarian self, shape voluntary aid workers' personal trajectories and “professional habitus” (p. 3). In addition, we examine the *doings* of the divide between guests and hosts and between migrants and volunteers by discussing specific sheltering practices, doing so by investigating what kinds of *labeling* are practiced and performed in the shelters that correspond to discourses upholding the divide between guests and hosts, pointing also to *practices of de-migranticization*, i.e., where seemingly natural orders of us vs. them are contested and to a certain degree subverted.

By moving beyond the divide, we work toward “interconnectedness,” as Tendayi Achiume elaborates in *Migration as Decolonization* (Achiume, 2019). Her analysis denounces the dogmatic logic of territorial nation-state sovereignty, and she refers to the marginalization of so many migrants today and fundamentally questions the state's right to exclude for “reasons tied to the distributive and corrective justice implications of the legacies of colonialism” (p. 1,517). Her critique of nation-states' sovereign right to exclude non-nationals is followed by a redefinition of sovereignty that acknowledges the political interconnectedness between former colonial subjects with the current “neocolonial empire” (p.1,520), proposing an approach to migration in which subjects from colonized nations are seen as “co-sovereign members (…) entitled to a say in the vehicles of effective collective self-determination” (p. 1,520). Similarly, Gilroy powerfully claims that “the little-known historical facts of Europe's openness to the colonial worlds …must be employed to challenge fantasies of the newly embattled European region as a culturally bleached or politically fortified space (Gilroy, 2004, p. 155–156).”

We see the transformative potential in approaching sheltering practices through interconnectedness. To specify this, we focus on spaces of conviviality. Conviviality, a notion that has different origins but relates in the first place to Gilroy's (2004) arguments on multicultural Europe and racial divides—articulates forms of cohabitation that transcend prescribed racialized social positions (see also Valluvan, 2016; Gutiérrez Rodríguez, 2020; Guadeloupe, 2022). In that sense, conviviality is the transformative power of renegotiating relations that emerge everyday—and in parallel to racialized structures—and that result in new forms of solidarity and social justice (Valluvan, 2016; see also Lapina, 2016). In the context of the shelters, however, we did not perceive conviviality as uncontested emergence that has the power to undo difference, as in the case of metropolitan dynamics and translocal culture discussed by Gilroy. We rather approach it as a constant struggle embedded in the relational practices of people looking for shelter as well as people looking to shelter. A struggle, nonetheless, may not undo differences and divides but does stretch and subvert its boundaries.

## 3. Methodological backstage approach

This study is framed in a project that aims to better understand the practices of two shelter organizations in Mexico and the Netherlands, by critically looking at the discourses and performativities of people interacting in them. We have used relational ethnography to qualitatively analyze the data collected in fieldwork periods between 2020 and 2022. Through this approach, the observations focused on the interactions of “two types of actors or agencies occupying different positions within the social space” bounded “together in a relationship of mutual dependence or struggle” (Desmond, 2014, p. 555); analyzing “fields rather than places, boundaries rather than bounded groups,” and “processes rather than processed people” (Desmond, 2014, p. 574). The observations are written from an autoethnographic and reflexive position (Denshire, 2014). In addition, the project relies on a combination of tools for ethnographic research, such as participatory observations and semi-structured and open interviews with a wide variety of actors. Informed consent was obtained from all the participants of this study, and this was documented via audio recordings.

In this study, we foreground reflexivity using a backstage approach (Aparna et al., 2020), which although it is not a direct reference to the well-known work of Goffman (1959), is inspired by a theater setting where artists preparing in the backstage look into the mirror before they perform. However, we acknowledge that the use of this metaphor in social research is not new. In his study with refugee communities, Miller (2004) reflects on his position as a researcher who is also an outsider, in relation to the access to these communities and the meaningfulness of the data collected. For that, he uses the “metaphor of frontstage and backstage behavior to illustrate both the complexity and importance of developing relational contexts that are based on trust” (p. 218), which he addresses as an outstanding “methodological issue in research with socially marginalized, politically oppressed communities” (p. 218). Despite Miller's valuable insights, his use of the backstage metaphor, in relation to Goffman's work, centers on questioning the degree of reliability of the information that participants provide to a researcher; hence, the importance of building trust to gain accessibility to such personal “backstage” where more authentic information can be found. Our use of backstage differs from Miller's, as we see the backstage as a space for academic reflexivity, where we can destabilize our privileges, recall our doubts and uncertainties, redirect our academic gazes, and seek for other ways of knowing (Aparna et al., 2020). In this study, the autoethnographic method is not only a data collection method but also a way of foregrounding the backstage as it allows for continuous problematization of the researcher situatedness, and his/her/their work in the overall processes of knowledge production.

Speaking from the methodological backstage aims to “critically re-look, (dis/re)engage, or deviate” from “dominant academic practices (…) one is trained in or expected to demonstrate expertise on” (Aparna et al., 2020, p. 111). Keeping “accountable to our own messy role in the messy processes of *re-search-ing*” in highly politicized fields of study allows us to critically reflect on the normalization of methods and “objects of inquiry” (Aparna et al., 2020) that might (un)wittingly contribute with border regimes and migration apparatuses. Backstaging our methodological choices helped us understand how power shapes our “fieldwork encounter(s),” the influence of our positionality in relation to the socio-political struggle over “what knowledges come to matter and why,” and the “direct links between academic work and border devices” (Aparna et al., 2020, p. 111). It inspired us to question the use of “migration” and “migration-related categories” and prevented us from taking volunteer or migrant as fixed and static categories.

Our analysis and writing style juxtaposes the experiences of people typically migranticized with those who are typically not. My (methodological) choice of working as a volunteer at both shelter organizations to embody such a role became particularly important for the auto-ethnographic insights, which I used as a research method, writing tool, and approach to dialogue with my own and others' experiences in “the field” (e.g., Denshire, 2014). Interestingly, this strategy helped me realize the multiple performativities embedded in my mobility processes, embodying the researcher who can move in and out of the field, the volunteer working for these organizations, and the migrant crossed by privilege and precarity. It is important to mention that in the process of writing, we experienced a constant tension between the analytic and descriptive parts of our texts. Even though we have defined “migrant” and “volunteer” as our analytical starting point, and even though we invented our terms such as “people looking for shelter” and “people looking to shelter,” the dispute over the meaning and usage of these words serves as an account of such “plasticity.” Similar to what happens within the shelters' dynamics, our writing shows the interchangeability of labels according to the specific part of the message we want to convey. Such multiplicity of terms helped us weigh our attempt to de-migranticize our narrative as an onerous one. We acknowledge that our writing choices might be overwhelming to some readers, but we hope that these readers take the shifts in terminologies as an invitation to reflect on the complexity that such effort to de-migranticize entails.

Although the organizations we worked with provide similar kinds of assistance to people on the move, their position in wider geopolitical landscapes of the migration regimes is rather different. Located close to the southern border of Mexico, Casa para Todes is part of a regional context particularly violent for marginalized populations, including the people crossing the border into the country without the authorization of the Mexican immigration authority. Since its creation, Casa para Todes has worked as a shelter providing “humanitarian assistance,” such as food, clothing, and basic medical aid, and as a “human rights center” advocating for the regularization of the people's migratory status. For over 10 years, they have sustained a clear politicized position in denouncing the violence(s) produced by the regional migration regime(s) and the abuses of the national and local immigration and law enforcement institutions.

Located at the eastern border of the Netherlands, Iedereen Welkom is part of a differently violent regional context. People staying in shelter organizations as Iedereen Welkom experience the violence of living without access to basic rights, such as housing, work, healthcare, and education. This relates to what Davies et al. (2017) and Mayblin et al. (2020) discuss as the “violent inaction” and “the slow violence of the everyday.” The priority of Iedereen Welkom is to provide a dignifying facility where “homeless undocumented refugees” (Iedereen Welkom's website 2020) can shower, eat, and stay for the night. In contrast with Casa para Todes, Iedereen Welkom's position, regarding the advocacy regarding people's oppression by the Dutch migration regime, seems less politicized. However, it is part of a network of organizations contesting in practice the effects of Dutch and EU's migration policy, denouncing at times, state projects that aim to normalize a narrative of forced return (Kalir and Wissink, 2016) for undocumented non-nationals, as it is the case for the *Landelijke Vreemdelingen Voorziening LVV* (The National Aliens' Facilities). Thus, an important aspect of our analysis of these organizations is the attempt to acknowledge the similarity in the dynamics making and unmaking the divide, and the structural “fabrics” in which such phenomena acquire significance.

## 4. Entangling trajectories: motives, interests, and privileges

Despite having moved (or not) across national borders in contrasting ways due to migration policies and border controls, the trajectories of so-called “migrants” and so-called “volunteers” entangle while interacting in the shelter's dynamics. In this section, we first present two vignettes that deliberately juxtapose people's experience at Casa para Todes and reflect on their need to be at the shelter, focusing on their motives “to look for” and “look to” shelter. Subsequently, we relate these needs to the question of geopolitical privilege. Aiming to de-migranticize our narrative through such reflections, we uncover the characteristic ambivalence of people's trajectories composed by both actors' need to experience the shelter, as they continue developing these and their careers.

### 4.1. De-migranticizing motives in the shelter

Vignette 1.

Andrés came to the shelter carrying almost no luggage, a few expectations and quite some knowledge of that place, it was the second time he was there. He had entered the country a couple of days before and needed information to know more about the current regional context. For that, he thought the best would be to stay at the shelter for some time. As he had done before, he kept planning the next steps in his journey, thinking on which paths to take, and imagining future possibilities and opportunities. He knew that once again, he would have to contribute to the shelter with work, both because he felt obliged to give something back but also because it felt right. After being interviewed by a shelter's worker, being explained and accepted the rules, he was allowed to enter. After resting from the journey, he started helping out with different chores, storing of food and cooking, cleaning, taking care of others, and making sure that people in the house followed the rules. He appreciated the help and information he received from the shelter's workers and the people that crossed borders like him. He did not mind much for sleeping in a room with many other people, eating the same food as everyone, waking up very early in the morning or negotiating different kinds of agreements with “the volunteers.” All of these things were worth the stay (Diary notes, February, 2018).

Vignette 2.

Cesar came to the shelter carrying light luggage, a few expectations and quite some knowledge of that place, it was the second time he was there. He had entered the country a couple of weeks before and needed information to know more about the current regional context, specifically about the work done at the shelter. For that, he thought the best would be to stay in it for some time. As he had done before, he kept planning the next steps in his journey, thinking on which paths to take, and imagining future possibilities and opportunities. He knew that working as a volunteer was demanding, but also that once again he would learn much from it, and also, it felt right. After being interviewed by a shelter's worker, being explained and accepted the rules, he was allowed to enter. After resting from the journey, he started helping out with different chores, logistics and organization of activities, cleaning, taking care of others, and making sure that people in the house followed the rules. He appreciated the help and information he received from the shelter's workers and the people he was supposed to help. He did not mind much for sleeping in a room with a few other people, eating the same food as everyone, waking up very early in the morning or negotiating different kinds of agreements with “the migrant people.” All of these things were worth the stay (Diary notes, April, 2021).

These vignettes juxtapose the contrasts and similarities regarding the mobility, motives, journeys, and activities inside the shelter, between a “migrant” and a “volunteer” living at Casa para Todes. Presented in vignette 2, I have volunteered two times at Casa para Todes at the same time I did my ethnographic work. Andrés, presented in vignette 1, stayed at the shelter also for the second time after having reached the northern border of Mexico and crossed into the United States, then for some reason, he went back to Honduras. Like me, Andrés too volunteered, working in the shelter's kitchen and the guard. We both embodied multiple roles and migrant stories at the very same time. During Andrés' stay, he spent time asking others about the safety of the route in the region, the current situation at the US and Mexico border-crossing, and the speed through which asylum cases were being received and resolved by the Mexican immigration authority. These moments of inquiry are indeed similar to those framed in my research as “data collection.” Interestingly, in this Mexican case, there is no limit to the amount of time so-called “migrants” are allowed to stay in the shelter. For so-called volunteers, there is a short stay of 2 months and a long stay of maximum 1 year. The short-stay volunteers live in the shelter, although they sleep in dormitories separated from the rest of the “migrant” guests. This temporal divide already indicates that questions of hosts vs. guests can work out in highly confusing ways as many of the “typical hosts” are actually passers-by.

By mirroring or juxtaposing these actors, we do not question the need for safety for some, and the specific expertise in terms of care of others. However, speaking with Malkki (2015) reveals a coeval, co-present neediness and interest situated on the “giving side” and the “receiving end” (p. 8). The fixation of both in a dichotomy of needs vs. interests reinforces the idea that migrants are exclusively driven by the need for something, *especially* to live, and that volunteers are exclusively driven by the interest to be involved and to discover more about something specific in the context where the shelter is situated. Such dichotomization happens at the level of the shelter's narrative, at the level of state migration policy, and at the level of academic knowledge production on migrant mobilities and sheltering practices. Instead, recognizing the specific motivations and neediness of both the “humanitarian aid recipient” and the “humanitarian benefactor” (Malkki, 2015) suggests the possibility of undoing the divide (as a distinction) between these subjects. Therefore, we argue that these vignettes help us acknowledge that, despite having aspects in common, both persons' experiences in the shelter are different mainly due to structural conditions inside and outside it.

It is the articulation (Laclau, 1996) of opposing discourses about “humanitarian benefactor” and humanitarian aid recipient” that occurs within shelters, that renders motives, needs, and interests as empty signifiers, which get a different—and often not acknowledged—meaning through the sheltering practices and performativities of the actors involved. Andrés and I had quite different experiences crossing the border and moving through Mexico, and yet we both did it, as well as we have both come back to the shelter and volunteered in it. In the end, the power imbalances produced by the migration regime have shaped our trajectories, as also the categories we have embodied. Due to the normalization of a narrative telling that migrants need the shelter, while volunteers are just interested in it, it is not possible to see that both actors need (to be at/to experience) the shelter, as much as they are interested in getting something from it.

### 4.2. Geopolitical privilege: trajectories and careers

Oscar and I met at Iedereen Welkom. He is a middle-aged man who came to the Netherlands partly due to the multi-layered violence he experienced in his country of origin, but also because he was “searching for a normal life” (diary notes, October 2020). He had lived in the shelter for at least 2 years. Back in his country, Oscar worked in the hospitality sector for a long time, but having no citizen service number (BSN) in the Netherlands, excluded him from accessing an education program that would validate his knowledge and professional experience. On December 2020, Oscar was expelled from the shelter due to a drug-related issue. He changed his phone number and practically disappeared. This situation made me question how discipline and control are enforced on the people that Iedereen Welkom and similar shelter organizations are supposed to care for. On September 2021, I heard from one of the persons staying at the shelter that Oscar was in Spain, where he had applied for asylum. In the meantime, he had been “allowed” by the local municipality in Spain to enroll in an education program that would certify his knowledge and work experience in the hospitality business. Whereas, his life in the Netherlands stagnated, over there, he was able to finish his education program, he lived in an apartment, and started looking for a job at a five-star hotel in just a few months (diary notes, April-June 2022).

In August 2020, I started working as a volunteer at Iedereen Welkom as part of my fieldwork activities. Different from my Dutch coworkers at the shelter, I am “allowed” to live in the Netherlands with a temporary residence permit which expires on the same date as my PhD contract. The process through which I arrived and then stayed in this country for almost 5 years has been determined by gender, ethnicity, and class markers (Amelina, 2021, p. 3) composing my “geopolitical privilege.” Identifying and navigating society as a cis-gender, mestizo man from Mexico City, has certainly shaped my trajectory as a “migration scholar,” but also as a “migrant,” having my mobility influencing my career, and vice versa. In 2018, after my graduation as a master's student, I extended my residence permit for 1 year, by paying the immigration authorities for a procedure called *zoekjaar* (orientation year). This procedure permits “highly skilled migrants” who have graduated from a higher education program at a Dutch institution to look for formal employment. I was able to apply to such a master's program by proving my English proficiency level through a *Toefl* test, something partly possible due to me and my parents' life-long investments in my education. What also helped me have a fluent domain of the English language was the fact that I had spent a couple of years working in the United States. In my early 20's, I traveled to this country with a tourist visa and worked “informally” in a restaurant, in precarious working conditions and having no access to social security. I am now considering applying for a permanent residence in the Netherlands to go on with my “career.”

For a variety of reasons, and coming from different contexts, Oscar and I ended up in the Netherlands ultimately looking to improve our lives. Our uneven life situations, in terms of privilege, determined the way we crossed borders, and the categories *ongedocumenteerde vreemdeling* (undocumented alien) and *kennismigrant* (highly skilled migrant) imposed on us by the Dutch state. Despite how migration regimes have governed our mobilities, we continued developing our careers as our trajectories unfolded. These life-story portraits show our passings between regularity and irregularity, precarity and stability, and mobility and immobility. Such passings are also moments in which we have been migranticized, in more privileged or unprivileged ways by the migration regime's apparatus. This structure has ultimately articulated our migratory status with the (in)accessibility to employment and education, “allowing” us to do, and be, just as much as our geopolitical privilege allows us. Even though this section puts central the micropolitics of the encounter of Oscar and myself in the shelter, it does address at the same time how geopolitical privilege can be understood as a constellation of political, cultural, and economic forces that place people in the shelter in particular ways. While acknowledging the influence that migration policy and architectures have in differentiating people's mobilities, this section emphasized the common places (the shelter), common drivers (motives and interests), and common aspirations and realizations (trajectories and careers) of people interacting at these shelter organizations.

## 5. Doing migration through administration and labeling

By starting from the idea that migration-related difference is an outcome of social practices, this section delves into two of them: administration and labeling. For the administration, we focus on the intake process for newly arrived people at Casa para Todes, relating it to the intake process at Iedereen Welkom. We describe the moment of the interview, framing it as a mechanism that reinforces the categories of “migrant” and “volunteer,” based on gender-, ethnicity-, and class-related differentiations. Subsequently, we discuss the practice of labeling. We do not only highlight how different labels are used, but we reflect on the effects they have on the daily interactions at the shelter.

### 5.1. The intake process

Vignette 3.

When they arrive to Casa para Todes, people looking for shelter are interviewed by a volunteer in a room where a desk separates the newly-arrived “guest” from the many times recently-arrived “host.” Volunteers are instructed to detect if the interviewee has experienced any kind of violence during their journey and evaluate the urgency of subsequent actions, especially if he/she/they have been injured, sexually assaulted, extorted or kidnapped, minding the level of distress and trauma such person might be experiencing. First, questions eliciting name, age, sex, nationality, place of birth, gender identity, mother language, religion, marital status, number of children, and trade or profession are asked. Then, questions eliciting the person's reasons for migrating, point of departure, border-crossing locations, means of transportation, time-line of their current journey, number and place of detentions by immigration authorities, and number and place of deportations. Finally, questions regarding encounters with security and containment forces like immigration agents, police and military corps, encounters with smugglers, and encounters with robbers and/or people who have harmed them in any way. Altogether, this information helps the shelter organization defining who are the people they host, how their trajectories look like, and to document and denounce the violence and abuses by state and non-state actors.

Vignette 4.

Before they arrive, people looking to shelter are required to fill out a form online, then a paid staff member of Casa para Todes interviews them. Since most of them live somewhere else in Mexico or abroad, the interviews happen mostly online. In the form, candidates are asked how they found out about the shelter and its volunteering service, whether they have previous experience in migration-related work or “vulnerable groups,” and what do they think they can contribute to, in terms of their abilities, knowledges, relevant experiences, hobbies, etc. At the interview, information regarding the candidate's level of study, professional formation and experience doing voluntary work, is discussed more in detail. Altogether, this information helps the staff member(s) responsible for selecting and managing the voluntary workers, to define who they are and where do they come from, as well as trying to foresee the impact of their work on the wellbeing of those hosted by the shelter.

These vignettes briefly describe the intake processes at Casa para Todes. It is important to acknowledge that the information elicited in the interview with so-called migrants helps to detect the circumstances in which each person arrives to better assist them. So it does for documenting changes in migration flows, people's containment and authority abuse by state actors, and the perpetration of crimes against people en route through Mexico. In this way, Casa para Todes contributes to a network of researchers and organizations advocating for the protection of “migrant populations” and broader changes in the migration policy (Wurtz, 2020). It is nevertheless problematic that the intake process for “the migrant” emphasizes the exceptionality of their mobility trajectory, being imposed by the state logics of criminalized migrations and following gender-, ethnicity-, and class-related markers. On the contrary, such markers are mostly overlooked in the intakes for volunteering candidates. Both intakes work on the assumption that the interviewees would fit either in one or the other role, to some extent identifiable as “the vulnerable” and “the care giver.” Despite its usefulness, the information elicited from people moving across borders at the margins of the migration architectures of the state turns the interview practice into a mechanism through which specific aspects of a person's life, mobility, and trajectory become migranticized, legitimizing a narrative that reproduces the figure of “the migrant.”

The intake process at Iedereen Welkom works similarly. The information elicited aims to identify the degree of vulnerability, mental and physical health condition, and the particular characteristics of the person's trajectory. It focuses on the person's mobility across physical borders and their experiences dealing with bureaucratic procedures within the EU's migration regime. With this information, the organization (represented by its coordinator) maps out a plan to channel their guest to other NGOs providing different services, mostly for accessing healthcare, legal aid, Dutch language courses, certain forms of education, and socialization within the community. The interview works as a mechanism that helps Iedereen Welkom create a profile that besides helping to address the person's situation also helps distribute the care and assistance work across the network of organizations. It is indeed important that such information is shared with these organizations; however, the narrative mobilizing the network emphasizes people's degree of vulnerability. In an interview, the coordinator of another organization providing housing to undocumented people commented: “in the end, the problem is that it is us, mostly Dutch people with papers, making the decisions of how people should be helped by us and the network, and which ways are better to assist people who don't get to decide on this” (Carmen, interview recording, June 2022).

During the interviews with both organizations before volunteering for them, I was never asked about my migratory status, how I had crossed borders to get there, or whether I had ever been detained or deported by an immigration authority. Moreover, I was never asked any information regarding my gender identity, ethnicity, and class, which are elements that have (and still do) determined my life and how I move across borders, matters that certainly keep shaping my trajectory. My “passings” between positions of “migrant” and “volunteer” have let me cross the boundaries dividing these categories in a seemingly fluid way. Yet, I ask myself: what makes me the migrant, the volunteer, and the researcher?

Next, we will discuss the power operating in the use of labels by the shelter organizations, concretely in the way these are used in communication and the effects they have in shaping people's reality.

### 5.2. Labeling and plasticity

Sheltering practices as the interviewing of newly arrived persons looking for shelter are legitimized by discourses of emergency attention, humanitarian assistance, and advocacy in the protection of people's rights. People living at Iedereen Welkom are addressed as *bewoners* (residents) by the volunteers and paid staff of the organization, mostly in their formal communications. This label is used internally in meetings and workshops, but also in different kinds of encounters with organizations in their network. Although the term “resident” stresses the fact that a person has an address and a place to live, it also relates to the state's authorization for someone to live in a country—i.e., someone holding a “residence permit.” Since the use of this term is mostly left to internal communications among staff members, people addressed as such might not be directly affected by it; however, it is important to note that in the daily relation with the so-called “residents,” some volunteers feel confronted by its usage, finding it contradictory and awkward to call people residents who lack the legal status of becoming a resident. Iedereen Welkom issues an ID card to each of its “residents” so they can identify themselves, mostly in case they are stopped by the police. On this study, the label *client* appears next to the person's name. Despite the usefulness of this card, preventing the escalation of an encounter with the police, the term client circumscribes the person in question to the shelter's materiality and discourse, as well as their political position in the community. This becomes relevant as the term client, often used by international and grassroots NGOs providing legal advice and access to healthcare to undocumented people, is also related to the developmental narrative of some humanitarian NGOs aiding migrants and refugees, through schemes based on assistance and services.

At Casa para Todes, the labels “migrant,” “asylum solicitant,” “refugee,” and “unaccompanied minor” are the most used in the communications within the organization and between this and other NGOs and state institutions. In the organization's context, these labels encompass the discourses related to the violence such people experience, having the possibility to report any crimes committed against them in Mexico with the help from the “human rights team” of the shelter. This procedure is important because it opens the possibility for someone to obtain a temporary regular migratory status recognized by the immigration authority, becoming then a “visitor for humanitarian reasons.” When reporting these events to the local authorities, volunteers usually use the term “victim of a crime” to refer to the person affected by them. In Mexico, the labels “migrant” and “victim” are commonly associated with the narratives of shelter organizations. It is a fact that the lives of people moving across the country at the margins of state controls are commodified via human trafficking, extorting, and diverse forms of exploitation (Vogt, 2013); however, the continuous association of specific (im)mobile populations to those labels provokes a simplification of the structural violence causing such commodification, hence the reduction of its manifold dimensions to migration-related aspects. Such reduction is visible as well in the use of labels, e.g., in the way the word victim is used as a prefix to describe complex mobility experiences, such as “victims of trafficking,” “victims of smuggling,” or “victims of extorting,” terms familiar to shelter organizations in Mexico. Contrastingly, it is not common to find the label “victim” in relation to the systemic/structural dynamics causing these kinds of experiences, e.g., “victims of state migration regimes,” “victims of neocolonial power structures,” or “victims of necropolitical policies.”

The labels used in sheltering practices often have multiple meanings, and some evoke stereotypical images around victimhood and migration. Interestingly, the organizations also carefully direct specific labels to specific audiences. Labels in sheltering practice do reflect a form of *plasticity*. DeBono (2019) refers to the plasticity in the use of words related to hospitality in southern Europe. DeBono draws on the notion of “plasticity” to appoint to the impoverishment of language at processes in which terms that might have had a specialized scientific origin, are “reimported to the vernacular” (p. 344), becoming vague and ambiguous, and holding multiple meanings. These plastic words can be seen as “floating signifiers” (Laclau, 1996) as the outcome of opposing and articulating discourses in which they are embedded and the meanings contained in them become disputed by different political groups competing to “assign their desired signified” (p. 345). Some of the labels presented above—such as resident and client—contain a series of contrasting signifiers (meanings) which form a “chain of equivalences,” “existing only in their differences to one another” (Laclau, 1996). At the moment in which a particular signifier dominates the others via a hegemonic process, assuming the representation of the rest, it becomes an “empty signifier” (Laclau, 1996, in DeBono, 2019). In such domination processes, certain groups gain power and hegemony through the use of specific labels, defining who can belong and who not (DeBono, 2019). As such, the label migrant can mean in particular contexts and cases that one belongs, while in a different discursive setting, it signifies non-belongingness.

In the Netherlands, for instance, the label “undocumented migrant” carries specific discursive characteristics related to a person's livelihood, such as homelessness, marginality, precarity, and vulnerability. Although people labeled as undocumented migrants many times lack a steady place to live and access to social services, and survive in precarious conditions, they might as well have paid work, attend education programs, and be active in different social groups for whom their knowledge and skills are highly appreciated. The use of labels does not always prevent someone from participating in social interactions outside the shelter, but gives place to social limitations provoked by the fixation on roles associated with labels as “undocumented migrant,” “refugee,” or “victim of trafficking.” This issue has implications for the way people experience a sense of place-making and belonging (Winters and Reiffen, 2019) while being indeed part of society. In terms of the differentiation made of so-called migrants from so-called volunteers, the plasticity of labels might contribute to the de-politization of the first, constraining or limiting their political agency in the shelter's structure. But what happens when labels are re-signified or contested? We focus on this potential in our next section.

## 6. Undoing the divide? Conviviality as passing and struggle

Having presented the migrant/volunteer divide, elaborating on people's motives, the entanglement of their trajectories, the mechanisms that categorize them, and the plasticity of the labels related to it, this section presents different moments and situations in which the divide has been challenged, contested, transgressed, and even undone by the people involved in sheltering practices. From these moments, we highlight *the passings* through which these actors have crossed the seemingly hard boundaries of the divide. Although passings are not seen as absolute and persistent forms of transgression, they do remind us about the potential for alternative relational politics at play. As an entry to this discussion, we present one auto-ethnographic illustration of shifting care and hospitality relations in the Mexican context:

Vignette 5

I was bitten by an insect in my right thigh while sleeping and in 2 weeks I could not move the whole leg. Lucrecia, who arrived at the shelter with her children and husband, and who also volunteered to coordinate the kitchen area, had seen me limping for a few days. She asked me what was the problem, and after I showed her the lump that appeared in my thigh, she advised me to burst it. She claimed that this kind of insect leaves larvae that eventually eat the flesh. I was very scared and went to three different doctors in the city, but despite having started with an antibiotics treatment, my leg was still hurting and the lump was still there. So 1 day, Lucrecia insisted that if I did not open that lump and clean inside it, the injure would get worse and the consequences might be bad. I trusted her, because she said that the same thing happened to her son and that after treating it he got better. We went to the infirmary, she laid me down on a stretcher and gave me something to bite on, then said “Cesar, hang on, it's going to hurt.” Only with the help of a syringe, a piece of cotton and alcohol she burst the lump and cleaned the wound, it was very painful indeed. After that happened I went to a different doctor to treat the wound. This person asked me who had treated my leg, I explained that I worked at the shelter and that it had been a woman staying there who insisted in “curing” my wound. The doctor told me to be grateful to Lucrecia because by the shape of the wound and my previous symptoms, it seemed that the insect's venom would keep on eating the tissue (Diary notes, July 2021).

In this illustration, I was literally the one being cared for, while I was not expected to be in that position. This experience shows a clear, however temporary, *passing* of roles and divides. The vignette therewith also represents how caring can become a “floating signifier” (Laclau, 1996) in all kinds of practices and moments inside the shelter where prescribed roles somehow disappeared: moments that vary from great laughter to shared mourning. However, to understand the transformative potential of these *passings*, we should not only look at relational dynamics within the spatial confines of the shelters. The context around these locations, as well as its internal (sheltering) processes related to rules and roles, produces a notable difference between what happens inside and outside its space. In other words, much of the hard work of undoing divides takes place in convivial spaces outside the shelter locations. Some people staying at the Casa para Todes, for instance, decide at times to leave (not to mention that they would sometimes be asked to leave) and rent a room in the city. As many of them had a good relationship with some of the volunteers, they often invite them over to eat and hang out. These interactions are important exactly because it transcends the rules and conditions of sheltering practices. These are—at least in potential—the unspectacularly meaningful moments of conviviality that emerge in parallel to sheltering conditions and indeed help to undo the prescribed social positions (Valluvan, 2016). Next to shared dinners, we see this potential unfolding in theme parks, football courts, and public squares. Although we feel that these moments are at the same time filled with active reflections and evaluations that directly and indirectly reaffirm the divide, we seek to acknowledge that they simultaneously signify shared grounds and new forms of solidarity that are formed in the everyday, as expressed in literature on conviviality. To make this insightful, we turn to the case of Iedereen Welkom.

Volunteers at Iedereen Welkom are the “contact person” of someone living at the shelter. Many of them, describe their one-on-one relationships with the people they accompany as genuinely based on friendship and trust. Both actors are acquainted with diverse aspects of each other's lives, many of which happen outside the shelter. In the following statements, volunteers tell about what they find important in these relations.

Maria:

I'm reflecting a lot about my role (working) with him, because I also see him as a friend and with friends you are motivating each other and trying to help each other, but I don't want to be his mother or his teacher, who is trying to tell him what to do, […] when I started with this internship at the Meldpunt Vreemdelingendetentie (Immigration Detention Hotline) he was also like, “ah Maria, you can handle that, because you have a lot more difficult situations to deal with in your head,” so he was really concerned about my mental situation and he was really glad that this internship is over right now (Interview recording, February 2021).

Koen:

There are just some guys I really have a good connection with and I think it's always nice to come in the house and just sit down and drink some tea and have a conversation, have a laugh, […] as a volunteer you are on their level (the residents), at least I'm trying to be there. Of course they think […] about you in some hierarchical way, but I'm just drinking tea and coffee with them and talking about bullshit all day and having a laugh. And this is the most important job of all the volunteers, I would say (Interview recording, February 2021).

Throughout the interviews and small-talk moments, these and other volunteers highlighted the importance of being recognized as a friend who is cared for by the people living in the shelter. Despite being constrained by the rules of the organization, these interactions are not strictly limited to the space of the shelter, as they continue unfolding in different spaces outside it. With time, both actors normalize the idea that the shelter is not a space excluded or disconnected from the rest of the community in the city and that their relationships with the volunteers are possible outside its materiality. By detaching from the shelter's materiality, people make room to acknowledge the person behind the role of the migrant and the volunteer. These are significant *passings* and they give room for consolidating deeper relationships. It is this commonality that can give way to contesting, destabilizing, or'rattling the cages”' of the dichotomous categorization and migranticization. Although it is difficult for both actors to fully get rid of the label/role they have been assigned through the shelter, these *passings* also point at what (Guadeloupe, 2022, p. 14) describes as conviviality: ceasing to be the totally separate other. In Guadalupe's case, this is an ethnic other, and in our case, it is the migrantizicised “other.” We refer to these *passings* certainly not as an antidote to fully undo the divide, nor as a total destabilizer, but as meaningful and momentous breaks that show us that other dynamics, other worlds, are possible.

Ceasing to be the “totally” and “essentialized” other though is often partial, signifies a struggle, as there is still a sense of being subordinated to the principles of the shelter. This struggle is something Koen refers to as being on “their level,” addressing the fact that both actors are subjected to the shelter's rules. When I asked Koen what were the difficulties of being a volunteer, he replied: “It was always a game between being a volunteer, applying rules and being strict, to being just myself […] it was always a clash between those two” (Interview recording, February 2021). Koen recalled a moment in which someone being sheltered asked him if he wanted to smoke marihuana with him, to which he responded: “ok, now I will switch to volunteer, then say like, no, we can't do this, you can't do this here” (Guadeloupe, 2022). Although it was not clear if the person wanted to smoke marihuana inside the shelter or not, and despite marihuana consumption being legal in the Netherlands, Koen immediately recurred to the logic of the shelter.

When I asked the same question about difficulties in her role as a volunteer, the young woman Josje responded: “Yeah, so, not the activities but the double role you have, because for me they feel as (being) friends, but you're also a volunteer, and that makes it really hard to say: so, I like you, but we can't do this together because it would not be appropriate in a way…” (Interview recording, February 2022). She elaborated by referring to a couple of times when she was asked if she wanted to go swimming or on a date by men living in the shelter, situations in which she would have felt “more vulnerable,” by exposing herself and her body to a group of men. The contrasts between Josje and Koen's experiences also tell about the role that gender plays in these interactions, something we have not addressed in this study, but which we believe should be furtherly analyzed.

Our findings indicate that people looking to shelter also look for—or struggle to find—convivial spaces to transgress the role-play of sheltering. In both settings under study, this search for new relational spaces also occurred in a broader sense. People related to the shelters co-organized with people from the community several cultural, artistic, and sport events, such as football matches, social cafés, and food and trade fairs. The intention behind these events is to facilitate moments in which all participants experience sharing, as the base for acknowledging and learning from differences and aiming to sustain stronger and more permanent processes of community building. Yet, the eventuality of these processes—as *special moments* to be advertised or *particular places* to be designed—immediately reflects their limitations in terms of conviviality (e.g., Lapina, 2016). To put it differently, the potentiality of Achiume's (2019) interconnectedness, or Gilroy's (2004) cohabitation, is easier to recognize in its straightforward form in political action. We have seen multiple moments whereby both people looking for shelter and people looking for shelter mingled and stood side-by-side. People interacting at Casa para Todes encountered as well in commemorations of International Migrants Day, the women's international struggle(s) on the 8th of March and demonstrations claiming justice for the 72 migrants found murdered in northern Mexico in 2010, also known as the San Fernando massacre. People interacting at Iedereen Welkom have encountered in demonstrations demanding a more humane asylum and migration policy, in places such as Moria, Calais, and Sarajevo, and more recently (September 2022), situating similar demands as part of a nation-wide protest sparked by the government's inaction affecting people waiting outside Ter Apel's asylum seeker center. In both the Dutch and the Mexican contexts, the people under study indeed interconnected in performing political action that demanded the end of racialized border logics that systemically stratify them, and others, in terms of their mobility rights. These are the moments where the “figure of the postcolonial migrant” is recognized as the anachronistic figure bound to the lost imperial past (Gilroy, 2004, p. 165). Even though we focused on analyzing the migrant/volunteer divide, mostly in relation to the micropolitics of sheltering practices happening inside and outside the shelter, we acknowledge the importance of studying the potential these moments of political solidarity have in undoing it. This we leave to our readers' consideration for further research.

## 7. Conclusion

This study investigates the extent to which sheltering practices contribute to the (un)doing of migration—i.e., the reproduction of migration-related difference. The insights regarding the *needs* related to the shelter, the administrative procedures, and labeling practices, as well as the *passings* and continuous *struggle over convivial relations*, articulate that shelters hold very specific mobility, political, and social relations for various periods of time. It is clear that shelters are strongly embedded in the wider architecture of migration governance, including its necropolitics and the further marginalization of underprivileged travelers (Davies et al., 2017). The way in which this embeddedness figures within in the different sheltering practices and performativities, however, varies. At times, this condition is articulated and reproduced by sheltering practices, as we have seen with the intake procedure and the plasticity of labels. The ambiguity produced by such plasticity in the form of “floating signifiers” opens up room in other moments, for questioning, contestations, and destabilization through different *passings*, as we particularly discussed in the section on potential conviviality. In our view, this conviviality does not necessarily result in a total transgression of the relational logics of the shelter. It does, however, “rattle the cages” of the categories that reproduce its underlying colonial design, the normalization of difference, and corresponding logic of “othering.”

With these reflections in mind, we would like to return to the question of de-migranticization as a form of knowledge production (Dahinden, 2016). The sheltering practices that we outlined are so dynamic that it is difficult to dichotomize them in terms of doing and undoing migration. Next to the use of state ingrained common-sense categories, for instance, we have come across highly creative and dynamic forms of categorization. Furthermore, the same people who perform the role of the guard or host look for conviviality in the relational politics. This is not just an argument to prevent dichotomized ideas, it rather implies that de-migranticization—as the disentangling of knowledge from presupposed and state-induced knowledge frameworks—is not only the work of academics (see also Amelina, 2022). In fact, it is part of the everyday relational struggles of shelter organizations, going beyond the relevance of discursive labels (how to call people) but entering instead the bodily emotions and social relations of people. In that sense, in terms of interconnection, there is much more to learn from the relations, conversations, knowledges, interconnectedness, and conviviality that are embedded in sheltering practices.

## Data availability statement

The data generated from this study contains elements that might identify its participants. Requests to access the datasets should be directed to CM-E, cesar.merlinescorza@ru.nl.

## Ethics statement

The study involving human participants was reviewed and approved by the Institutional Review Board of Radboud University. Informed consent was obtained from the participants and documented via audio recording.

## Author contributions

CM-E collected and analyzed the data used for this paper and wrote the vignettes and first drafts of the manuscript. JS and TD contributed with active writing and reviewing of all sections. All authors built the argument and contributing with their own analytical insights. All authors contributed to the article and approved the submitted version.

## Footnotes

^1^For both shelters, we prefer to use pseudonyms.

^2^Even though we acknowledge the notion of race as a socially constructed one, we highlight the fact that for various actors embedded in migration regimes and architectures, race is used as a marker by the Mexican National Migration Institute's (INM) agents, who detain people moving across borders based on racial profiling.
